# Evaluating the Compatibility of the Digit-in-Noise Test with Hearing Screening in Individuals with Intellectual Disabilities: A Pilot Study

**DOI:** 10.3390/diagnostics14111202

**Published:** 2024-06-06

**Authors:** Noa Shmerler, Ronit Saban-Bezalel, Leah Fostick

**Affiliations:** 1 Department of Communication Disorders, Ariel University, Ariel 4070000, Israel; noashmer@gmail.com (N.S.); ronitsa@ariel.ac.il (R.S.-B.); 2 Auditory Perception Lab in the Name of Laurent Levy, Ariel University, Ariel 4070000, Israel

**Keywords:** hearing screening, intellectual disability, digit-in-noise test

## Abstract

Hearing impairment among adults with intellectual disability (ID) is notably prevalent yet frequently underdiagnosed due, in part, to the challenges associated with traditional hearing screening methods in this population. This study explores the effectiveness of the Digit-in-Noise (DIN) test as a viable alternative for hearing screening within natural settings and with familiar personnel. A total of 16 Hebrew-speaking adults with ID were recruited from supported employment programs, 10 of whom completed the study. The DIN test, which was administered in a daily environment using a simple digital device, evaluated the speech recognition threshold in noise. Results indicated that while some participants performed comparably to typically developing individuals, others showed varying levels of hearing thresholds, suggesting diverse auditory capabilities within the ID population. This pilot study confirms that the DIN test can be feasibly integrated into routine care settings, offering a friendly and accessible method for assessing hearing abilities in adults with ID. The findings advocate for the broader adoption of and potential modifications to the DIN Test to enhance its applicability and inclusiveness, thereby improving diagnostic accuracy and subsequent auditory care for this underserved population.

## 1. Introduction

Hearing loss is a prevalent medical condition among adults with intellectual disability (ID), affecting about 30% of this population [1,2]. Hearing loss can stem from various factors, such as congenital anomalies, excessive cerumen, and genetic syndromes [3,4,5]. This condition is crucial for day-to-day functioning and can impede the development of language and communication skills. It is also linked to cognitive, emotional, and social declines, impacting family relationships and quality of life [6,7,8,9]. Despite a heightened risk for hearing loss among individuals with ID, studies suggest they are underdiagnosed.

Hearing loss underdiagnosis for individuals with ID arises from the lack of accessibility to hearing screening [1,10,11,12] or factors related to the test and testing procedure. Thus, relating hearing loss to ID-related behaviour, having teachers unskilled in detecting hearing loss and audiologists unskilled in communicating with an ID population, prioritizing other, more visible medical issues (diagnostic overshadowing), and requiring calm and cooperating behaviour in an unfamiliar and sometimes stressful environment (a sound-proof booth), cause the underdiagnosis of hearing impairment in the ID population. Therefore, there is a need for hearing tests that can be administered in a daily, natural environment by familiar personnel.

The Digit-in-Noise (DIN) test [13,14] was developed as a screening test and requires the examinee to repeat sequences of three digits (such as 2-3-4) presented against masked background noise. The test measures the threshold for recognizing speech in the noise (the speech recognition threshold in noise, SRTn), which is determined as the signal-to-noise ratio (SNR), i.e., the difference between the speech volume and the noise volume in which the digit sequences were detected in 50% of the cases. This reflects the daily challenge of understanding speech in a noisy environment, making the test appropriate for hearing screening.

Several characteristics of the DIN test suggest it can be suitable as a hearing screening test for people with ID. First, using digits, a familiar concept from everyday life for people with ID, makes it simple to relate to. Second, it is friendly and accessible, does not require special equipment, and can be carried out using mobile phones, computers, or computer boards (tablets); thus, it can be performed by teachers or staff members who know the individual. Third, since the test is presented with background noise, there is no need for a special soundproof room, and it can be administered in the examinees’ natural, everyday surroundings. Fourth, it has a relatively short administration time, thus making it appropriate for people with short sustained attention and poor cooperation. Therefore, the present study aimed to examine the applicability of the DIN test, a simple, accessible, and friendly tool, as a hearing screening test for people with ID.

## 2. Materials and Methods

### 2.1. Participants

**Figure 1:**
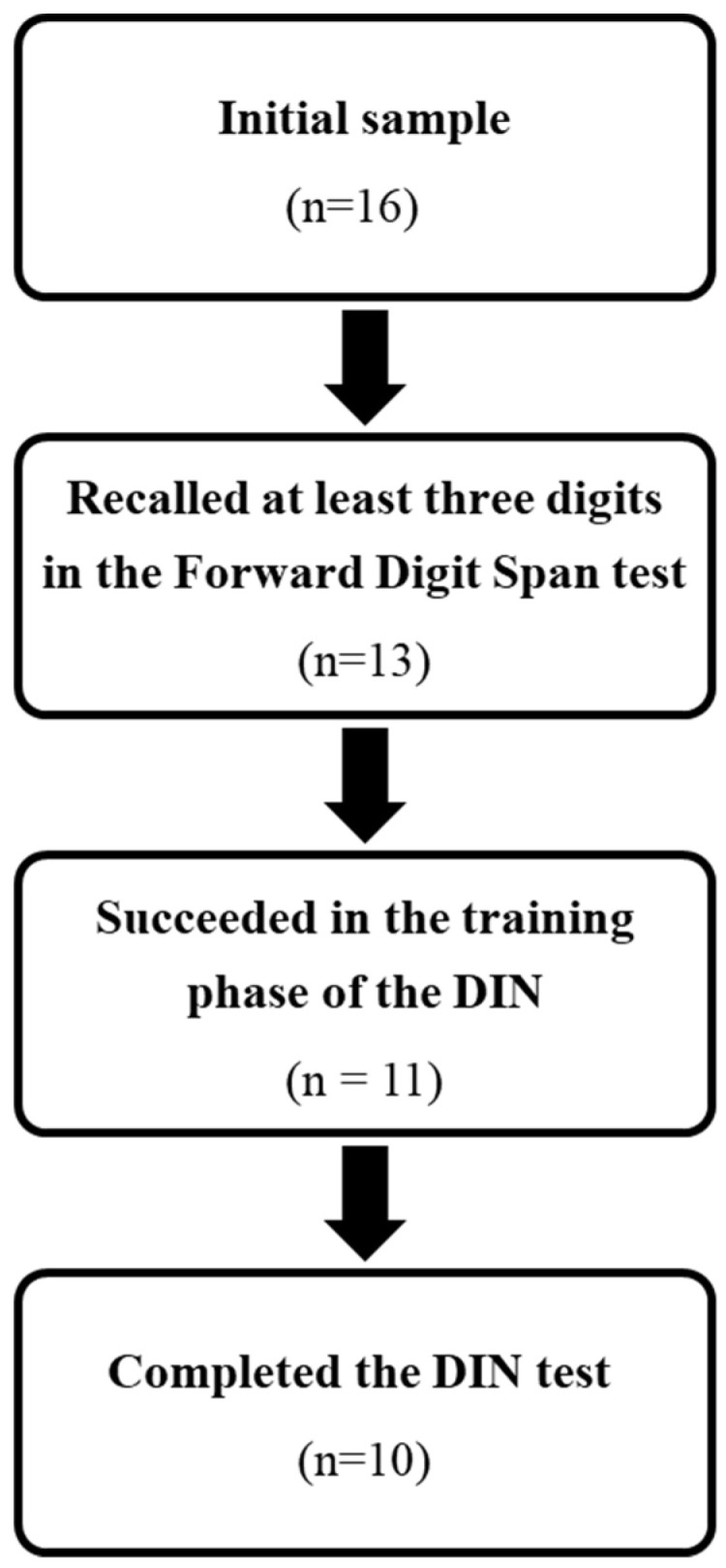
The recruitment process.

Sixteen participants (five women) aged 21–40 years (mean = 27.75, S.D. = 6.54 years) were recruited from supported employment programs. All were Hebrew speakers diagnosed with ID, according to the Diagnostic Committees of the Ministry of Welfare and Social Services (Table 1). The criteria for inclusion in this study were (1) recognizing the numbers 0 to 9; and (2) recalling at least three digits in Wechsler’s Forward Digit Span test [15]. All participants met the first criteria, but three recalled only two digits and were therefore excluded from the study. Of the 13 participants included in the study, two failed the training session when background noise was delivered, and one did not complete the test, leaving 10 participants who completed the study procedure (Figure 1).

### 2.2. Screening Tests

In the Forward Digit Span [15] test, participants were asked to repeat a series of digits (from 2 to 9 digits) exactly as they were presented. Trials increased in length by one digit if one or more trials of a particular length were completed correctly until two trials of the same length of digits were completed incorrectly (Table 1).

In a semantic comprehension test (The Psycholinguistic Assessments of Language Processing in Aphasia, PALPA-47, [16,17], participants were asked to point to one picture out of five that matched a word presented orally. The test included 40 target words (Table 1).

### 2.3. Hearing Tests

The Digit-in-Noise (DIN) test, Hebrew version [18], obtained a mean threshold of −9.7 (sd = 0.9 dB) from TD young adults. The test included 25 number triplets that were presented to both ears with a background speech-shaped-noise of the digits, using an iPad IOS8 (Apple Inc., Cupertino, CA, USA) device attached to TDH-39 headphones (Telephonics Corporation, Huntington, NY, USA). The volume of the noise was fixed across the test, and the volume of the digits changed according to the participants’ responses (decreasing following a correct response and increasing following an error). The participants responded by repeating the numbers aloud, and the examiner typed the answer (instead of the usual self-administration). The test started with a training session including six trials, three with a fixed SNR of 15 dB and three with changing SNRs following correct or incorrect answers at step size of 5 dB. Following the training phase, the test started with an SNR of 0 dB and varied adaptively following a one-up one-down procedure, with a step size of 2 dB. The SRTn was calculated as the average SNR of the last ten trials.

The Audiometry Hearing Screening test, a standard pure-tone audiometry test, was conducted using an MA28 audiometer and TDH39 headphones. The test measured the air-conduction hearing thresholds of each ear at 500, 1000, 2000, and 4000 Hz. The thresholds were categorized as good (25 dB or less in all frequencies in both ears), borderline (30 dB in at least one of the frequencies tested), or poor (35 dB or more in at least one of the frequencies tested). The criterion for borderline hearing was set because the test was administered in a quiet but not soundproofed room. The participants heard each tested frequency at a gradually decreasing volume and were requested to raise their hand when they heard the sound.

### 2.4. Assessment Scales

Participants were assessed on a 1 to 5 scale to determine the level of adaptation required during the audiometry and DIN tests, their ability to understand the instructions of the DIN test, and the effect of the presence of noise on the participants (Table 2).

### 2.5. Procedure

The universit and the Ministry of Welfare and Social Services Institutional Review Boards approved the study. The first author (N.S.), an audiologist and speech–language pathologist experienced in working with people with ID, administered all the tests. The participants’ parents each signed an informed consent form for their child’s participation in the study. The participants signed an adapted informed consent after receiving explanations about the study’s aims and procedure. The study was conducted in a quiet room at the participants’ employment center and was carried out in one to two 1-h meetings, depending on the participant’s needs. Participants were first screened for digit span, followed by the two hearing tests conducted randomly, and then the semantic comprehension test. When all tasks were completed, the participants received a coffee-and-pastry voucher to the local cafeteria.

## 3. Results

### 3.1. Hearing Tests

Table 3 shows the thresholds in the two hearing tests obtained by the 10 participants who completed the DIN. In the DIN test, four participants had thresholds like those obtained by TD individuals (−7.8 dB and lower), three had moderately higher thresholds (−4.6 to −7 dB), and an additional three participants had much higher thresholds than those of the TD group (+1.4 to −3.4 dB). In the audiometry test, three participants had good hearing (20–25 dB), three had borderline hearing (30 dB), and four participants had poor hearing (35 dB or more).

### 3.2. Adaptation and Response Levels

The results of the adaptation level are presented in Table 4. In the audiometry test, one participant did not need assistance (score 1), and four needed mild assistance (score 2). The other eight participants needed marked assistance (scores 3 and 4). In the DIN test, four participants answered continuously throughout the test without stopping (score 1), and six needed additional assistance from the examiner (scores 2, 3, and 4). Three participants failed to repeat the digits (score 5) and did not complete the test.

The response level scales test showed that most of the participants understood the instructions of the DIN test quite easily. Seven participants understood the instructions without assistance (score 1) and six participants did not understand the instructions initially but understood them during the training (scores 2, 3, and 4). Four participants did not refer to the noise (score 1), and three mentioned its presence (score 3). Six participants were disturbed by the noise (score 5), two of whom dropped out because of it.

## 4. Discussion

The aim of the current study was to test whether the DIN test can be used for hearing screening among people with ID. The participants fully cooperated with the test, partly due to feeling comfortable in their natural daily environment and partly due to having the test administered by a professional staff member specialized in the ID population. Such a professional knows how to motivate individuals to participate in the test and mediate it appropriately. The test device, a tablet, is very accessible and familiar to people with ID, and the participants in the study like engaging with it. Thus, this pilot study showed that the unique factors relating to the test and its setting make it compatible with this population, although some adaptation may be required.

The participants’ hearing was also evaluated using an audiometry test. Such a behavioral hearing test is challenging for people with ID, and the use of additional hearing tests and objective measures is recommended [19]. Since the present study was preliminary and focused on the DIN test’s compatibility with people with ID rather than assessing their hearing thresholds, we avoided using additional tests, especially objective measures that can feel intrusive. To overcome behavioral challenges, the researcher who tested the participants was experienced in working with people with ID and made necessary adaptations to the test according to their special needs. Nevertheless, comparing the data from the two hearing tests was difficult since the norms in both tests were obtained from the TD population and thus did not consider the special needs of people with ID and the adaptations they require. As such, the current study’s data analysis provides a complex picture that cannot be explained by the individuals’ ID level or semantic comprehension as they were homogeneous in their ID level (mostly mild) and their level of semantic comprehension (ten had a semantic comprehension score of 77.5–97.5%, and three had lower scores of 62.5–65%).

Some participants had comparable thresholds in both the DIN and audiometry tests (for example, participants #9 and #10); some participants showed better performance in the DIN test than in the audiometry test (for example, participants #1 and #2); and some participants were better in the audiometry test than the DIN test (for example, participant #3). This incompatibility between tests could result from difficulty responding to the audiometer’s abstract pure tone sound relative to the familiar digits of the DIN or difficulty remembering the three digits in the DIN test. Indeed, all participants with a minimal score in digit span (a score of 3) also had a low DIN threshold. However, this pilot sample is too small to draw a definite conclusion on this topic.

Of the thirteen participants who performed the DIN, nine related to its noise, six of whom stated it was disturbing. Still, most of the participants (ten out of thirteen) kept performing the test despite the noise, and only two out of thirteen dropped out because of it. The adaptation scale showed that most participants needed at least some level of adaptation in both the DIN and audiometry tests. This aligns with the widely accepted practices in the clinical management of ID in which adaptation enhances overall functioning. The adaptation provided in the present study allowed almost all participants to complete the tests. In general, the DIN test’s advantages regarding the test and its setting make it a hearing screening tool compatible with people with ID. However, its application is currently limited since it requires a memory span of at least three digits. In addition, as with other hearing tests, it lacks feedback during the test. Such feedback can serve as a mediating and motivational factor. Therefore, we suggest that a modified version of the DIN test will respond to these shortcomings; it will enable the repetition of only two digits, thus enlarging the range of participants that can perform the test and better reflecting their hearing performance rather than their memory span. It will also provide feedback following each trial (like the feedback given in the training session). Such modifications to the DIN test will make the test friendly and accessible and will provide adapted mediation for people with ID. This, in turn, will enable the proper diagnosis and rehabilitation of hearing impairment, improving people with ID’s daily functioning.

## Figures and Tables

**Table 1:** The characteristics of the 16 participants recruited for this study.

Participant#	Gender	Age	The Level of Intellectual Disability (ID)	Digit Span ^1^ (WAIS-IV)	Semantic Comprehension (PALPA-47)
1	Male	24	Mild to moderate ID	4	77.5%
2	Male	23	Mild ID; Fragile X	3	90%
3	Female	25	Mild ID	3	85%
4	Male	38	Mild ID	4	62.5%
5	Male	22	Mild ID	5	92.5%
6	Female	23	Mild ID	4	62.5%
7	Male	39	Mild ID	3	82.5%
8	Male	30	Moderate ID	4	97.5%
9	Male	25	Mild ID	6	92.5%
10	Male	40	Moderate ID	4	65%
11	Male	22	Mild ID	4	90%
12	Female	23	Mild ID; Down syndrome	3	85%
13	Female	25	Mild ID	2	92.5%
14	Male	21	Mild to moderate ID; Down syndrome	2	85%
15	Male	31	Mild to moderate ID; Fragile X	2	67.5%
16	Female	33	Mild to moderate ID	3	92.5%

^1^ Raw scores. ID = Intellectual Disability

**Table 2:** Description of assessment scales used in the study.

Score	Definition
Adaptation scale for DIN test:	
1	No assistance needed. The participant always responds as instructed (reporting the digits or guessing when they are too quiet to perceive);
2	Some assistance is needed. The participant mostly responds as instructed but occasionally needs some encouragement to respond (*“did you hear the numbers? guess if not”*);
3	Assistance is needed. The participant responds as instructed, but when the digits are too quiet, they sometimes need encouragement to respond.
4	Extreme assistance is needed. The participant responds as instructed, but when the digits are too quiet, they always need much encouragement to respond;
5	Does not answer at all.
Adaptation scale for audiometry hearing screening:	
1	No assistance needed. The participant always raises their hand in response to the sound as instructed.
2	Some assistance needed. The participant mostly responds as instructed but occasionally does not raise their hand and is asked to respond verbally instead (to say *“I heard it”*).
3	Assistance is needed. The participant always responds verbally, but mostly spontaneously after hearing the sound. Sometimes there is a need to prompt the response and ask, *“did you hear the sound?”*
4	Extreme assistance is needed. The participant responds only after being asked, *“did you hear the sound?”*
5	Does not answer at all.
Level of response to the DIN test:	
1	Understands the instructions easily before initiating the training;
2	Understands the instructions after one training trial;
3	Understands the instructions after two training trials;
4	Understands the instructions after three or more training trials;
5	Fails to understand the test instructions.
The effect of noise:	
1	The participant does not refer to the presence of the noise;
2	
3	The participant mentions the presence of noise;
4	
5	the noise is very disturbing to the participant.

**Table 3:** Thresholds of the DIN test and the audiometry hearing screening for 10 participants who completed the DIN test.

Participant#	DIN SRTn	Audiometry Threshold (Pure Tone)								Hearing Average	Hearing Level Category
		Right Ear				Left Ear					
		0.5 Hz	1 kHz	2 kHz	4 kHz	0.5 Hz	1 kHz	2 kHz	4 kHz		
1	−9.8	30	25	20	5	30	25	10	20	20.62	borderline
2	−5	25	20	45	50	0	15	10	45	26.25	poor
3	+1.4	15	15	25	25	25	25	25	25	22.5	good
4	−2.6	30	35	20	20	30	25	25	35	27.5	poor
5	−7.8	15	20	15	10	20	20	20	20	17.5	good
6	−8.2	25	15	10	35	30	20	20	25	22.5	poor
7	−4.6	30	25	10	25	25	20	5	15	19.37	borderline
9	−8.2	30	25	10	5	25	20	10	0	15.62	borderline
10	−3.4	30	35	45	40	30	35	50	30	36.87	poor
11	−7	20	20	10	5	25	25	15	25	18.12	good

**Table 4:** Levels of adaptation and response for the hearing screening tests.

	Adaptation		Response	
Participant#	Audiometry Screening	DIN Test	Understanding Instructions	Effect of Noise
1	3	1	1	1
2	3	1	1	1
3	3	4	2	3
4	4	4	3	3
5	2	3	1	1
6	4	3	1	3
7	3	2	3	1
8	4	5	1	5
9	2	3	1	5
10	4	1	2	5
11	1	1	1	5
12	2	5	4	5
16	2	5	4	5

## Data Availability

All the data collected in this pilot study is published in the manuscript.
